# Band-like transport in highly crystalline graphene films from defective graphene oxides

**DOI:** 10.1038/srep28936

**Published:** 2016-07-01

**Authors:** R. Negishi, M. Akabori, T. Ito, Y. Watanabe, Y. Kobayashi

**Affiliations:** 1 Department of Applied Physics, Osaka University, Suita 565-0871, Japan; 2 Center for Nano Materials and Technology, JAIST, Nomi 923-1292, Japan; 3 Synchrotron Radiation Research Center, Nagoya University, Nagoya 464-8603, Japan; 4 Graduate School of Engineering, Nagoya University, Nagoya 464-8603, Japan; 5 Aichi Synchrotron Research Center, Seto, 489-0965, Japan

## Abstract

The electrical transport property of the reduced graphene oxide (rGO) thin-films synthesized from defective GO through thermal treatment in a reactive ethanol environment at high temperature above 1000 °C shows a band-like transport with small thermal activation energy (*E*_a_~10 meV) that occurs during high carrier mobility (~210 cm^2^/Vs). Electrical and structural analysis using X-ray absorption fine structure, the valence band photo-electron, Raman spectra and transmission electron microscopy indicate that a high temperature process above 1000 °C in the ethanol environment leads to an extraordinary expansion of the conjugated π-electron system in rGO due to the efficient restoration of the graphitic structure. We reveal that *E*_a_ decreases with the increasing density of states near the Fermi level due to the expansion of the conjugated π-electron system in the rGO. This means that *E*_a_ corresponds to the energy gap between the top of the valence band and the bottom of the conduction band. The origin of the band-like transport can be explained by the carriers, which are more easily excited into the conduction band due to the decreasing energy gap with the expansion of the conjugated π-electron system in the rGO.

Utilizing a carrier transport that reflects the intrinsic graphene band structure is essential for excellent electrical performance of graphene-based transistors. Graphene oxide (GO), which is produced by the oxidation of graphite, has attracted great interest in the large-area synthesis of graphene owing to cost-effectiveness and mass production. Since GO has rich oxygen-containing groups, much effort has explored such reduction processes as thermal^1^,^2^,^3^,^4^ and chemical reductions^5^,^6^,^7^. The most straight-forward goal of the reduction process is to produce graphene-like materials that resemble the highly crystalline graphene prepared by the mechanical exfoliation of graphite crystal. Although these processes enable us to efficiently remove the oxygen-containing groups, it is difficult to repair such defects as vacancy and an amorphous-like π network composed of a mixture of *sp*^2^ and *sp*^3^ in the GO produced by the oxidation of graphite.

Su *et al*. demonstrated that field effect transistors (FETs) using a single rGO flake prepared by thermal treatment in a reactive ethanol environment (hereafter ethanol treatment) show high conductivities and carrier mobilities^8^. The excellent electrical performance is caused by the efficient restoration of the graphitic structure in a single rGO flake from the observation of Raman spectroscopy. However, their work fails to mention the carrier transport mechanism is the dominant factor for operating devices. Moreover, since the FET using a single rGO flake in their work is not suitable for electrical device applications, it is important to elucidate the electrical transport properties in rGO thin-film (not a single flake) transistors (TFTs) in the large-area beyond the size of a single flake. It is necessary to develop the fabrication process of large-area graphene thin films showing the intrinsic electrical transport properties from defective GO thin films. Unfortunately, the electrical performance of large-area GO thin-films reduced by conventional process degrades more than that of a single rGO flake because of carrier scattering at the interface among rGO flakes^5^,^9^.

In previous work^10^, we found that ethanol treatment at a process temperature up to 950 °C has a remarkable suppression effect on carrier scattering among rGO flakes in large-area films. We also revealed that the carrier transport mechanism in large rGO films is described by 2-dimensional variable range hopping (2D-VRH) conduction observed in amorphous and organic semiconductors^11^,^12^. The observed 2D-VRH conduction through the localized states in the energy gap originating from electron confinement^13^ means that many defects remain in the rGO. For device applications utilizing the electrical performance of the intrinsic graphene material with ultra-high carrier mobility, it is necessary to obtain large-area graphene films that show band-like transport (not VRH conduction). In this study, we found that a high temperature process above 1000 °C in ethanol treatment enables us to overcome this issue from the analysis of carrier transport properties, X-ray absorption fine structure (XAFS), valence band photo-electron spectra observed from rGO films, Raman spectra and transmission electron microscopy (TEM).

## Results

### Evaluation of carrier mobilities of rGO films

**Figure 1:**
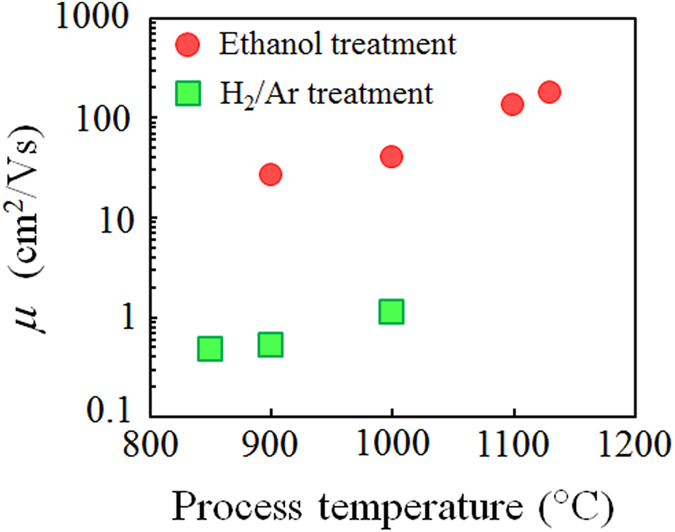
Evaluation of carrier mobilities of rGO films prepared by thermal treatment in reactive ethanol and inert H_2_/Ar gas environments. Carrier mobilities (*μ*) of rGO films prepared by ethanol 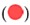 and H_2_/Ar treatments 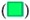 as function of process temperatures. Data in *μ* evaluated from rGO films prepared by H_2_/Ar treatment at 900 and 850 °C, reported previously^10^.

Figure 1 shows the carrier mobilities of the rGO films prepared by thermal treatment in reactive ethanol and inert H_2_/Ar gas environments (hereafter, H_2_/Ar treatment), indicated by red circles and green squares, respectively. The carrier mobilities of the rGO films prepared by ethanol treatment as a function of the process temperatures are evaluated from Hall-effect measurements using the van der Pauw method. The geometry of the rGO films in the van der Pauw device is a square, several hundred micrometers on a side, this is considerably larger than the average size of the single GO flake (~1 μm) used in our experiments, indicating that thin-film includes the interface among single rGO flakes. The carrier mobilities of the rGO films prepared by H_2_/Ar treatment are evaluated from the source-drain current (*I*_*sd*_) as a function of the gate voltage (*V*_*g*_) in TFTs using a standard formula^14^. We could not observe the Hall voltage in the rGO films, which were prepared by H_2_/Ar treatment due to low conductivity and electrical non-uniformity. The carrier mobilities improved with increasing process temperature in each treatment. Note that the carrier (hole) mobilities in the rGO films prepared by ethanol treatment show significantly higher values than in the rGO films prepared by H_2_/Ar treatment. The highest observed mobility reached ~210 cm^2^/Vs at room temperature (~270 cm^2^/Vs at 77 K), which is higher than the carrier mobilities (0.06–95 cm^2^/Vs) of the rGO films prepared by other reduction methods^5^,^15^,^16^,^17^,^18^,^19^. The ethanol treatment at a high process temperature effectively improved the carrier mobility of the rGO films.

**Figure 2:**
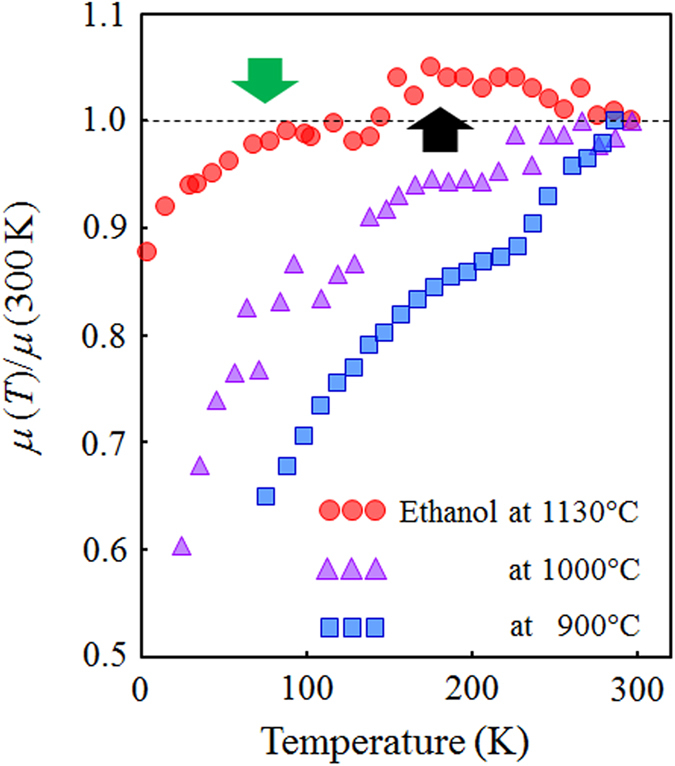
Temperature dependence of carrier mobilities (*μ*) of rGO films. The rGO films are prepared by ethanol treatment at 1130 °C (red circles), 1000 °C (purple triangles) and 900 °C (blue squares), respectively. *μ* is normalized by a value of *μ* at *T* = 300 K.

The temperature dependence of the carrier mobility is a convenient way to identify the dominant factors of such carrier scattering as phonons, structural defects, and charge impurities^20^,^21^. Figure 2 shows the temperature dependence of the carrier mobilities (*μ(T)*) of the rGO films prepared by ethanol treatment. To clearly exhibit the temperature dependence, *μ(T)* is normalized by the carrier mobilities measured at 300 K. The values of *μ*(*T*)/*μ*(300 K) observed from the rGO films at a process temperature below 1000 °C decrease with a decreasing measurement temperature, which is a typical behavior of 2D-VRH conduction through localized states^11^,^12^. The phenomenon is frequently observed in the GO films prepared by thermal and chemical reduction^22^,^23^,^24^. In 2D-VRH conduction, since the carrier jumps from one localized state to another in a large energy gap occurring in the electron confinement due to the small *sp*^2^-hybridization domains in rGO^13^, the carrier mobility and conductivity are very small compared with those observed from mechanical exfoliated graphene.

The behavior of *μ*(*T*)/*μ*(300 K) observed from the rGO prepared by ethanol treatment at a high process temperature of 1130 °C is much different; the value of *μ*(*T*)/*μ*(300 K) shows a maximum value at around 160 K, as indicated by the large black arrow in Fig. 2. The increasing value of *μ*(*T*)/*μ*(300 K) with decreasing measurement temperature from 300 to 160 K indicates which dominant factor of carrier scattering becomes phonon scattering^20^. We observed continuous decreasing carrier concentration with decreasing measurement temperature (see Supplementary Fig. S1). In general, carrier mobility decreases with decreasing carrier concentration. However, from 300 to 160 K, the carrier mobility increases with decreasing carrier concentration. This is caused by the suppression of phonon scattering^25^ and indicates the band transport. We also observed a fall of carrier mobility in a low-temperature region below ~60 K, compared with the *μ*(300 K), indicated by the large green arrow in Fig. 2. This means that VRH conduction is a dominant factor in the carrier transport mechanism under the low-temperature region. Unfortunately, the transition temperature from VRH and the band-like transport cannot be correctly determined from the temperature dependence of the carrier mobilities because the carrier mobility includes the effect of the change in the carrier concentration in addition to the carrier transport mechanism. Next we focus on the temperature dependence of the carrier transport mechanism.

### Temperature dependence of conductivity of rGO films

We measured the temperature dependence of the conductivity to identify the carrier transport mechanism. In the 2D-VRH model, the temperature dependence of the conductivity (*σ*(*T*)) has the following form^11^


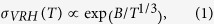


**Figure 3:**
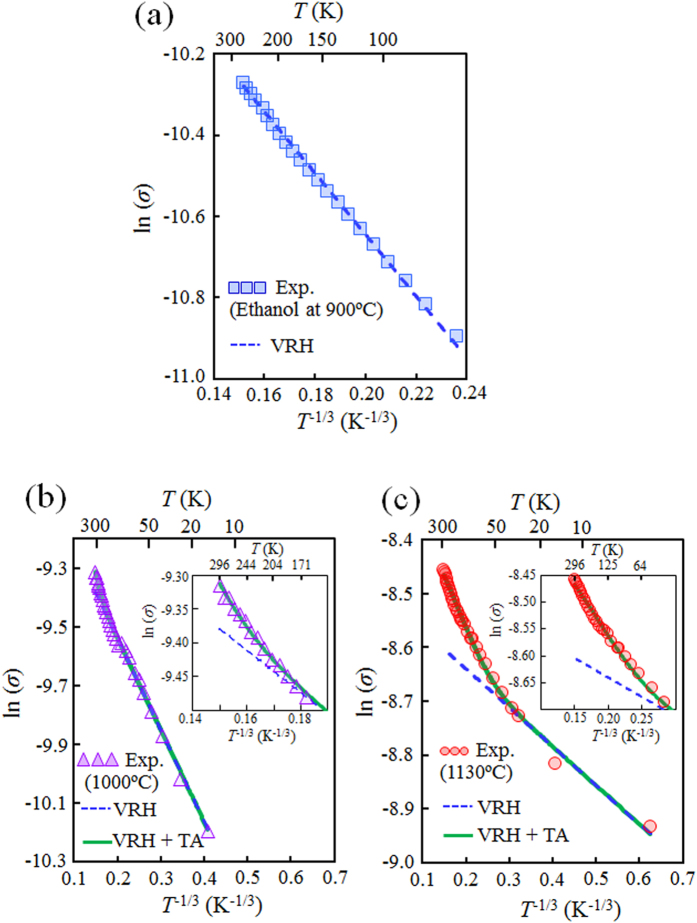
Analysis of conductance in rGO films. ln(*σ*) vs. *T*^−1/3^ are observed from rGO films prepared by ethanol treatment at (**a**) 900, (**b**) 1000 and (c) 1130 °C. Experimental data are fitted by 2D-VRH (blue dashed lines) or the sum of TA and 2D-VRH (green solid line) conductions.

Hopping parameter *B* is expressed as


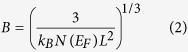


where *k*_B_ is the Boltzmann constant, *L* is the localization length of the electronic wave function for the conjugated π-electron system in the rGO, and *N*(*E*_F_) is the density of states (DOS) near the Fermi level. Figure 3 shows a plot of ln(*σ*) as a function of *T*^−1/3^. The ln(*σ*) measured in the rGO films prepared by ethanol treatment at 900 °C is well-fitted by the 2D-VRH conduction (Fig. 3(a)). On the other hand, we could not obtain well-fitting curves for the ln(*σ*) measured at the rGO films prepared by ethanol treatment at a process temperature above 1000 °C with Eq. (1). Similar complicated behavior in the temperature dependence of the conductivity is observed in a well-reduced single GO flake (not thin films), and G. Eda et al. pointed out that a combination of hopping and thermal activation at the midgap states in the rGO contributes to the carrier transport properties^26^. As shown in Fig. 2, the contribution of the thermally activated (TA) conduction reflects the fact that phonon scattering^25^ is observed in the rGO films prepared by ethanol treatment at high process temperature above 1000 °C. The characteristics of ln(*σ*) measured from the rGO films prepared by ethanol treatment at the high process temperature (Fig. 3(b,c)) can be fitted by the sum of the 2D-VRH and TA conductions (green solid line), expressed by


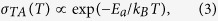


The carrier transport properties that show a combination of the 2D-VRH and TA conductions are commonly observed in disordered systems, such as amorphous semiconductors in bulk materials^27^. This is the first observation of carrier transport properties explained by the sum of the 2D-VRH and TA conductions in graphene thin-films synthesized from GO as single-atomic-layer materials. The contribution of TA conduction in the carrier transport mechanism suggests that the band-like transport occurs by forming continuous conduction/valence bands. Note that the TA conduction is observed at low temperature until ~60 K in the rGO films prepared by ethanol treatment at a high process temperature (1130 °C), and the limited temperature at ~60 K is lower than that at ~170 K, observed in the rGO films by ethanol treatment at a lower process temperature (1000 °C) (Fig. 3). Therefore, our findings indicate that a higher process temperature above 1000 °C is a key technique for the synthesis of large-area graphene thin films that show the intrinsic electrical properties of graphene from defective GO materials.

From fitting analysis, we can statistically estimate *L* and thermal activation energy (*E*_a_) as shown in Table 1. *E*_a_ is defined as the energy required for exciting carriers into non-localized states beyond the mobility edges^27^. The higher process temperature in ethanol treatment increases *L*, and this trend agrees with the results in our previous report where the process temperature was limited to 950 °C^10^. Note that the values of *L* in the rGO films increased extraordinarily by ethanol treatment at a higher process temperature than 1000 °C. *E*_a_ decreases with an increasing process temperature from 1000 to 1130 °C. Since an increasing *L* denotes the expansion of the conjugated π-electron system in the rGO, it is expected that the origin of *E*_a_ is the electrical structures with non-localized states due to the formation of large *sp*^2^ domains composed of the conjugated π-electron system. Then we focused on the change in the electrical structures of the rGO films through various thermal treatments.

### Evaluation of electrical structures of rGO

**Figure 4:**
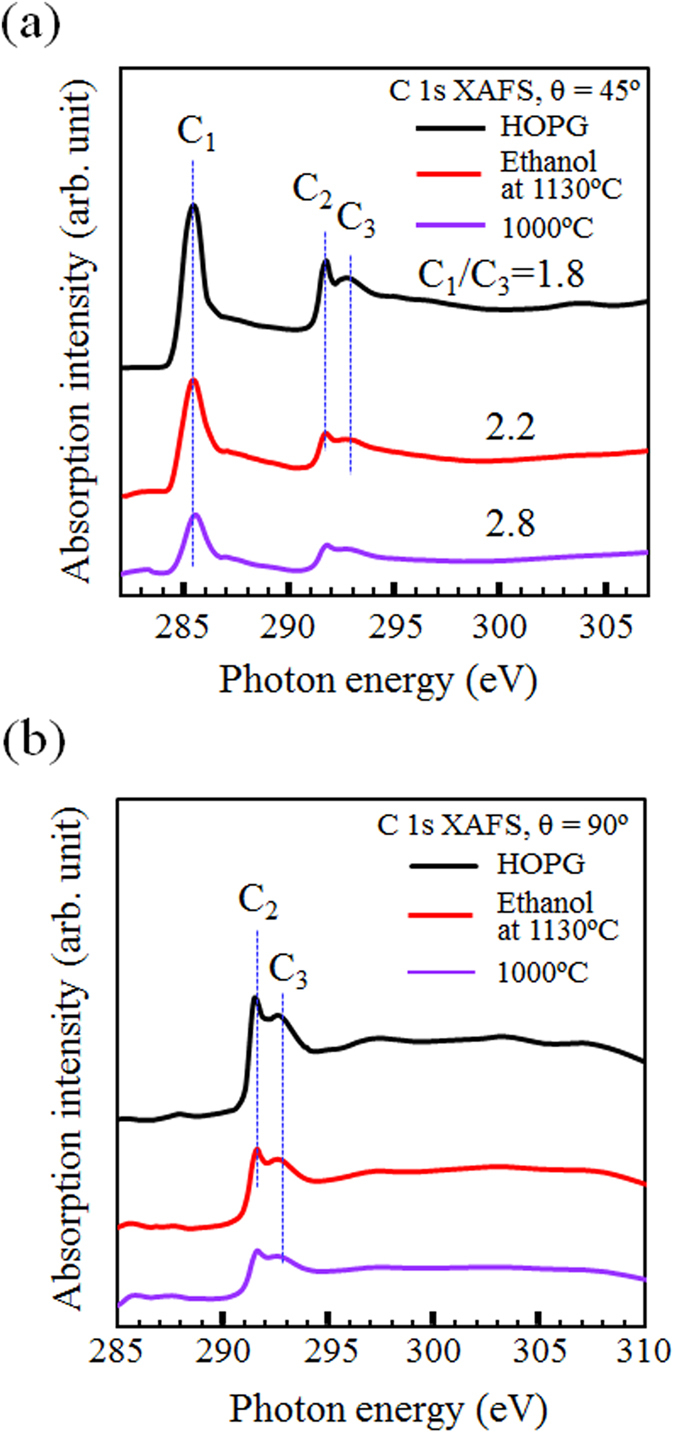
X-ray absorption fine structure spectra (XAFS). XAFS spectra are observed from HOPG (black line), rGO films prepared by ethanol treatment at 1130 °C (red line) and 1000 °C (purple line) with incident angles of (**a**) 45° and (**b**) 90°, respectively.

X-ray absorption fine structure (XAFS) is a powerful technique for investigating unoccupied electron structures. Figure 4 shows the C K-edge XAFS spectra observed from rGO films prepared by ethanol treatment at 1130 °C (red line), 1000 °C (purple line) and highly ordered pyrolytic graphite (HOPG: black line) as a reference. The spectra are normalized by the incident beam intensity measured simultaneously using the photoelectron yield of a clean gold grid located upstream from the analysis chamber. Features C_1_ and C_3_ at approximately 285.5 and 292.7 eV in the XAFS spectra with two different incident angles (45 and 90°) are assigned to the transitions from the C 1s to the unoccupied π^*^ and unoccupied σ^*^ states, respectively^28^,^29^. The observed C_1_ feature vanishes for the incident angle of θ = 90° because the π^*^ state with an out-of-plane orbital in the graphene plane is perpendicular to the electric field vector of the X-rays. The intensity ratio of C_1_ and C_3,_ observed from the rGO prepared by ethanol treatment at a high temperature (1130 °C), approaches that observed from HOPG rather than that from the rGO films prepared by ethanol treatment at a lower process temperature (1000 °C). Moreover, the XAFS spectra observed from the rGO films prepared by ethanol treatment at a high temperature show a sharp peak at 291.7 eV, originating from an excitonic state^30^,^31^ labeled by C_2_ in Fig. 4, indicating that ethanol treatment at high process temperature of 1100 °C leads to the formation of highly crystalline graphene.

### Structural restoration of graphitic structure in rGO flakes

**Figure 5:**
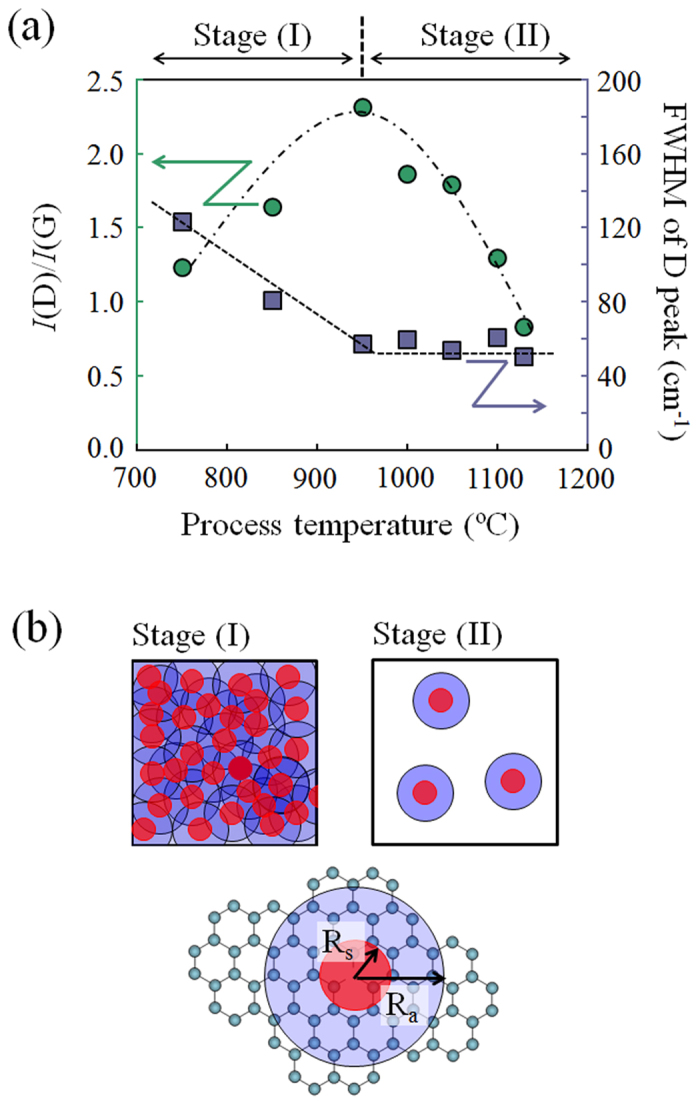
Evaluation of defects in rGO films. **(a)** *I*(D)/*I*(G) and FWHM of D peak evaluated from Raman spectra observed from rGO prepared by ethanol treatment as a function of process temperatures (see Supplementary Fig. 3). **(b)** Red circles with radius of *R*_*s*_ are structurally disordered areas, and light blue circles with radius of *R*_*a*_ are area surrounding defect points, leading to observation of D-band peak^36^,^37^.

In the previous section, we revealed that ethanol treatment at a high process temperature above 1000 °C improves the crystallinity of the graphitic structures and expands the conjugated π-electron system in the rGO. We also confirmed that high-temperature treatments in inert or reactive gases are effective to remove oxygen-containing functional groups, as previously reported^8^,^32^,^33^ (see Supplementary Fig. S2). In this section, we focus on the structural defects in the remaining rGO through various thermal treatments. The intensity ratio of the D-band (~1350 cm^−1^) and the G-band (~1580 cm^−1^) in the Raman spectra is useful to evaluate the structural restoration of the rGO^8^,^34^,^35^. Figure 5(a) shows the *I*(D)/*I*(G) and FWHM of the D-band peak (Γ_D_) evaluated from the Raman spectra as a function of the process temperature during ethanol treatment (see Supplementary Fig. S3). L. G. Cançado *et al*. experimentally demonstrated that the value of Γ_D_ decreases with a deceasing distance between defects (L_D_) in disordered graphene structures with L_D_ below ~5 nm, and shows a constant value for structures with L_D_ above ~5 nm^36^. The D-band intensity given by the ethanol treatment at 950 °C shows a maximum value; *I*(D)/*I*(G) increases as the process temperature increases to 950 °C (Stage (I)) and decreases for process temperature *T *> 950 °C (Stage (II)). This behavior can be explained by modeling so that the defect region on the graphene sheet is composed of two circular areas measured from the defect point (Fig. 5(b))^36^,^37^. One circle with a radius of *R*_*s*_ (red regions) is the structurally disordered area, and the other described as *R*_*a*_ (>*R*_*s*_) is the area surrounding the defect point in which D-band scattering takes place. The lattice structure is preserved in the area, but the proximity to a defect causes a mixing of Bloch states near the *K* and *K*’ valleys in Brillouin zone of the graphene. This leads to an enhancement of the intensity of the D-band due to breaking of selection rules^36^,^37^. We call this region the activated region (blue area in Fig. 5(b)). For the low density of the defects in the rGO that correspond to Stage (II), the total area of the activated region is proportional to the number of defects. However, for the high density of the defects corresponding to Stage (I), where the *L*_*D*_ is shorter than difference *R*_*a*_-*R*_*s*_, the activated regions start to overlap and eventually saturate. We observed that the *sp*^3^ structures still remain in the rGO prepared by a lower process temperature below 950 °C in spite of the ethanol vapor environment (see Supplementary Fig. S4). On the other hand, the *sp*^3^ structures dramatically decrease through ethanol treatment at higher process temperature above 1100 °C. This means that it is difficult to reconstruct the conjugated π-electron system (*sp*^2^ network) from an amorphous-like π network composed of a mixture of *sp*^2^ and *sp*^3^ structures. The healing such defects as an amorphous-like π network, domain boundaries and vacancies does not proceed under low process temperature (see Supplementary Fig. S6), although the oxygen-containing functional groups are efficiently removed at a lower process temperature below 950 °C. In this case, there is hardly a difference of L_D_ between the rGO and the pristine GO except for the density of the oxygen-containing functional groups. Since the L_D_ is shorter than the difference *R*_*a*_-*R*_*s*_ in the pristine GO, the activated regions in the rGO prepared at process temperature increasing until 950 °C still overlap between them. As a result, the *I*(D)/*I*(G) shows a maximum value (Fig. 5(a)). From these characteristics, we can obviously see a decreasing amount of defects in the rGO films prepared by ethanol treatment at temperature above 950 °C.

We also observed that the *I*(D)/*I*(G) characteristics, obtained from the rGO films prepared by Ar/H_2_ treatment at a high process temperature (1100 °C), are almost the same as those from the rGO films prepared by ethanol treatment at a lower process temperature below 950 °C, corresponding to Stage (I) (see Supplementary Fig. S5). The changes in the *I*(D) suggest that the structural restoration due to healing the defects efficiently proceeds by the ethanol treatment at a higher process temperature above 1000 °C^38^.

As more direct evidence for the structural restoration, atomic structures of the suspended monolayer rGO sheet are observed using TEM (see Supplementary Fig. S6). In the rGO prepared by ethanol treatment at low process temperature at 900 °C (Supplementary Fig. S6(a)), the many defects such as an amorphous-like network composed of a mixture of *sp*^2^ and *sp*^3^ structures are observed in the whole region. On the other hand, in the rGO by ethanol treatment at high temperature (1100 °C), the periodic spots are observed indicating improved graphene crystallinity. This indicates the crystallinity of the rGO is dramatically improved by the ethanol treatment at higher process temperature above 1000 °C. These features are in good agreement with the results of Raman spectra as shown in Fig. 5 and Supplementary Figs S3, S4 and S5. The process temperature in ethanol treatment is near 1000 °C, where the carrier transport mechanism changes from 2D-VRH conduction to a combination of 2D-VRH and TA conductions (Fig. 3). This means that removing the oxygen-containing groups is inadequate to observe the TA conduction in the rGO films; we must restore the conjugated graphene structure (*sp*^2^ network) from the defective GO because the extraordinary expansion of the conjugated π-electron system due to structural restoration leads to TA conduction.

## Discussion

A process temperature below approximately 950 °C in the ethanol environment is insufficient for the formation of the conjugated π-electron system in rGO (Fig. 5 and Supplementary Fig. S6), although the condition enables the efficient removal of the oxygen-containing functional groups^8^,^32^,^33^ (see Supplementary Fig. S2). Considering that the contribution of TA conduction with *E*_*a*_ is observed in the rGO films prepared by ethanol treatment at a process temperature above 1000 °C (Fig. 3), the origin of TA conduction depends on the change in the band structures near the Fermi level with the structural restoration of the graphitic structure in the GO.

**Figure 6:**
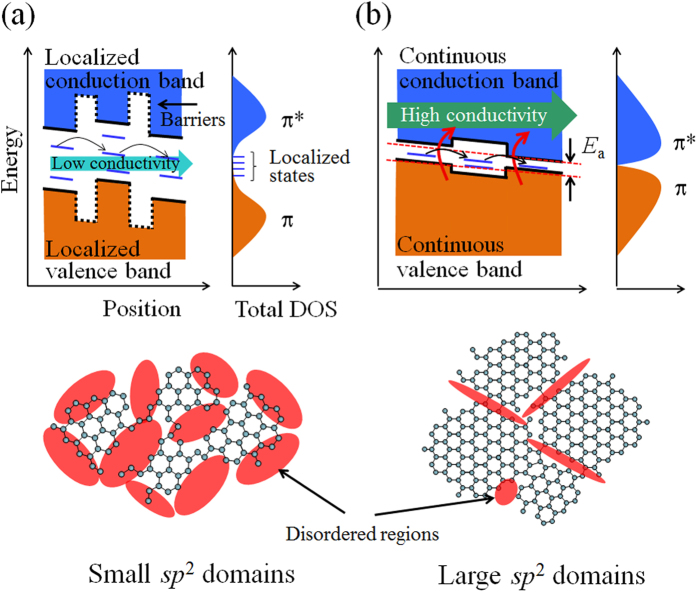
Carrier transport mechanism of rGO films. The valence band (orange region) and conduction band (blue region) structures for the rGO with (**a**) small *sp*^2^ and (**b**) large *sp*^2^ domains are illustrated. For small *sp*^2^ domain, energy levels quantized by electron confinements have large energy gap, and carriers pass through localized states in the energy gap by hopping process (black arrows). Since large *sp*^2^ domain reduces energy gap and vanishing potential barrier among domains, high conductivities of rGO are observed where carriers are more easily excited from continuous valence band to continuous conduction band at room temperature.

When the restoration of the graphitic structure in the rGO is insufficient due to a low process temperature, small domains composed of *sp*^2^- hybridization carbon atoms with a large energy gap due to electron confinement^13^ are formed. Since the small *sp*^2^ domains are surrounded by the large disordered regions composed of point defects and *sp*^3^ domains, the barrier height among the small *sp*^2^ domains is higher than the thermal energy at room temperature. The isolated quantum dots or localized states lead to the formation of electron and hole puddles near the charge neutrality point^39^. In this case, the carrier transport requires high energy to excite the carriers from the continuous valence band to the conduction band. As a result, the carrier transport properties are described by the 2D-VHR model through the localized states in the large energy gap (Fig. 6(a)).

**Figure 7:**
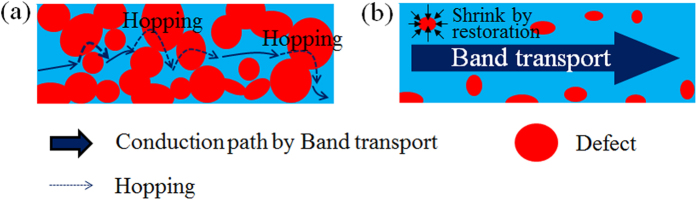
Illustrations of conduction path model of the rGO films with (**a**) high density of defects below percolation threshold, and (**b**) low density of defects above percolation threshold. In the case of **(a)**, carrier transport mechanism is dominant by hopping model. In the low density of defects as **(b)**, the carrier conduction shows the band-like transport by the formation of continuous and large *sp*^2^ domains in the rGO.

The formation of large *sp*^2^ domains due to the efficient restoration of the graphitic structure in the rGO flake yields an interesting insight into the carrier transport mechanism. Since the expansion of the conjugated π-electron system in the rGO leads to a vanishing potential barrier among domains by decreasing disordered regions, continuous conduction and valence band structures with a small energy gap are formed, as shown in Fig. 6(b). Although the defects slightly remain in the rGO as shown in Fig. 5 and Supplementary Figs S2, S3 and S4, it seems that the conduction path passes through the continuous *sp*^2^ domains and avoids the high potential barrier due to the defects as schematically illustrated in Fig. 7. The localized states suggest that increasing DOS near the Fermi level and in the rGO prepared by the higher temperature treatment under the ethanol environment can be found in the valence band spectra measured by angle-resolved photo-electron spectroscopy (see Supplementary Fig. S7). In this case, the carriers can be more easily excited from the top of the continuous valence band to the bottom of the continuous conduction band at room temperature (red arrows). At the low temperature region where the thermal activation energy is smaller than the energy gap, 2D-VRH is the dominant mechanism in the carrier transport properties (black arrows) (Fig. 3(b,c)). The *E*_*a*_, evaluated from the fitting analysis of the conductivity, is equivalent to the average energy gap originating from the electron confinement, as shown in Fig. 6(b). The combination of TA and VRH conductions is also observed in the highly crystalline bilayer graphene placed under a perpendicular electric field^25^. This is the first observation of carrier transport properties showing band-like transport in graphene thin films synthesized from defective GO materials.

In conclusion, thermal annealing in an ethanol environment at high temperature above 1000 °C provides highly crystalline graphene with extremely high carrier mobility in the rGO films from defective graphene oxide. The carrier transport mechanism of rGO films shows a band-like transport rather than 2D-VRH at room temperature. The improvement of the electrical performance of the rGO films is caused by the formation of continuous band structures in the rGO due to the efficient structural restoration of crystalline graphene with an extraordinary expansion of the conjugated π-electron system. These results indicate that ethanol treatment at high temperature is a key technology to produce large-area highly crystalline graphene thin-films similar to mechanical exfoliated graphene from defective graphene oxide.

## Methods

### Preparation of graphene oxide thin films on SiO_2_/Si substrate

Uniform GO (aqueous GO solution, Graphene Laboratories Inc.) thin-films are prepared by an electrostatic self-assembly method^6^, where the surface of the SiO_2_(280–320 nm)/Si substrate is modified by 3-amino-propyltrimethoxysilanne (APTMS). Since the GO flakes contain negatively charged functional groups such as carboxylic acid and phenolic hydroxyl group when dispersed in water^7^, they can be adsorbed onto positively charged amino-terminated SiO_2_/Si substrate by immersing the substrate into GO aqueous dispersion. Since the electrostatic adsorption between the substrate and GO is stronger than physical adsorption among the GO flakes, the extra GO flakes on the first GO layer can be easily rinsed off in pure water. From the observations of atomic force microscope, we confirm that the rGO films after the thermal treatments are formed as a continuous thin-film whose thickness is 1–3 layers by overlapping the single rGO flakes.

### Reduction and restoration of graphene oxide films

The reduction and restoration of GO films were carried out by the thermal treatment in reactive ethanol and inert H_2_/Ar gas environments at 750–1130 °C using a chemical vapor deposition (CVD) apparatus in a furnace^10^. Ethanol was used as carbon feedstock to repair defects in the rGO films^8^. A typical restoration and reduction process of GO films is as follows: (1) The GO films on the SiO_2_/Si substrate were preheated at 600 °C in a H_2_(3%)/Ar gas flow. (2) The furnace temperature was regulated to the prescribed temperature: 750–1130 °C. (3) After reaching the prescribed temperature, ethanol vapor at 0.5 standard cubic centimeters per minute (sccm) was introduced with H_2_/Ar as a carrier gas at 150–250 sccm. If the ethanol flow rate exceeds the rate used here, amorphous carbon is deposited on the whole sample surface due to the production of reactive species by the thermal decomposition of carbon feedstock in the gas phase^40^.

### Characterizations and electrical measurements of rGO films

X-ray Photoelectron Spectroscopy (XPS) measurements were performed using a kratos AXIS-Ultra spectrometer with monochromatic Al Kα X-ray radiation at the Japan Advanced Institute of Science and Technology. The unoccupied and occupied electronic states of the rGO films and HOPG were evaluated from X-ray adsorption fine structure (XAFS) spectra and angle-resolved photoelectron spectra using synchrotron radiation on beam line BL-7U at the Aichi Synchrotron Radiation Center. Raman spectra were acquired at room temperature using 532-nm laser excitation with a 100 x objective lens (typical spot diameter ~1 μm). The carrier mobilities and conductivities of the rGO films were determined by employing Hall-effect measurements in the van der Pauw configuration using the Bio-Rad HL5500PC system. TEM observations were carried out using JEM-ARM200F (JEOL Ltd.) Electron energy is 60 keV.

## Additional Information

**How to cite this article**: Negishi, R. *et al*. Band-like transport in highly crystalline graphene films from defective graphene oxides. *Sci. Rep.* **6**, 28936; doi: 10.1038/srep28936 (2016).

## Supplementary Material

Supplementary Information

## Figures and Tables

**Table 1:** *E*_*a*_ and *L* evaluated from fitting analysis of the conductivity.

	H2/Ar treatment at 1130 ºC	Ethanol treatment at 900 ºC	1000 ºC	1130 ºC
Ea/meV			84.6	16.3 ± 4.0
L/nm	4.3 ± 1.0	6.3 ± 1.3	30	184.0 ± 39.4
Transport Mechanism	VRH	VRH	TA+VRH	TA+VRH
Number of data	3	5	1	4

We statistically evaluated the *E*_a_ and *L* as average values from several experimental data except for the data obtained from the rGO films prepared by ethanol treatment at 1000 °C.
