# Supplementary material for: Band-like transport in highly crystalline graphene films from defective graphene oxides
Source: Sci Rep. 2016 Jul 1;6:28936. doi: 10.1038/srep28936 (PMC4929449; doi:10.1038/srep28936)
Supplement: Supplementary Information [file srep28936-s1.pdf]

Supplementary figures

Title:

Band-like transport in highly crystalline graphene films from defective graphene oxides

R. Negishi[1,*], M. Akabori[2], T. Ito[3], Y. Watanabe[4] and Y. Kobayashi[1]

[1]*Department of Applied Physics, Osaka University, Suita 565-0871, Japan*
[2]*Center for Nano Materials and Technology, JAIST, Nomi 923-1292, Japan*
[3]*Synchrotron Radiation Research Center, Nagoya University, Nagoya 464-8603, Japan*
[4]*Aichi Synchrotron Research Center, Seto, 489-0965, Japan*

*Corresponding author; Tel: **+81**-6-6879-4684, Fax: **+81**-6-6879-7863
E-mail address: negishi@ap.eng.osaka-u.ac.jp (R. Negishi)

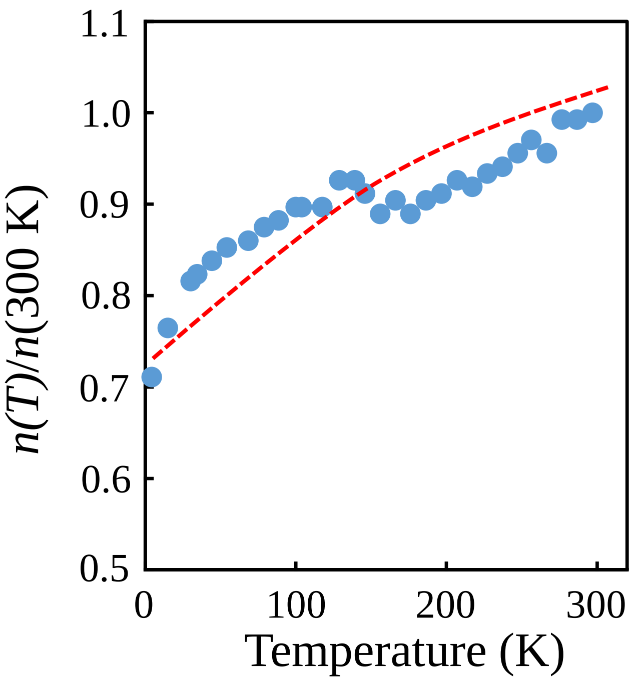

Supplementary Figure S1: *Temperature dependence of carrier concentration (n) observed from the rGO films prepared by ethanol treatment at 1130ºC.* The data (●) are simultaneously obtained with measurements of the carrier mobility as shown in Fig. 2. Red dashed line is an eye guide. The *n* is normalized by a value of *n* at *T*=300 K.

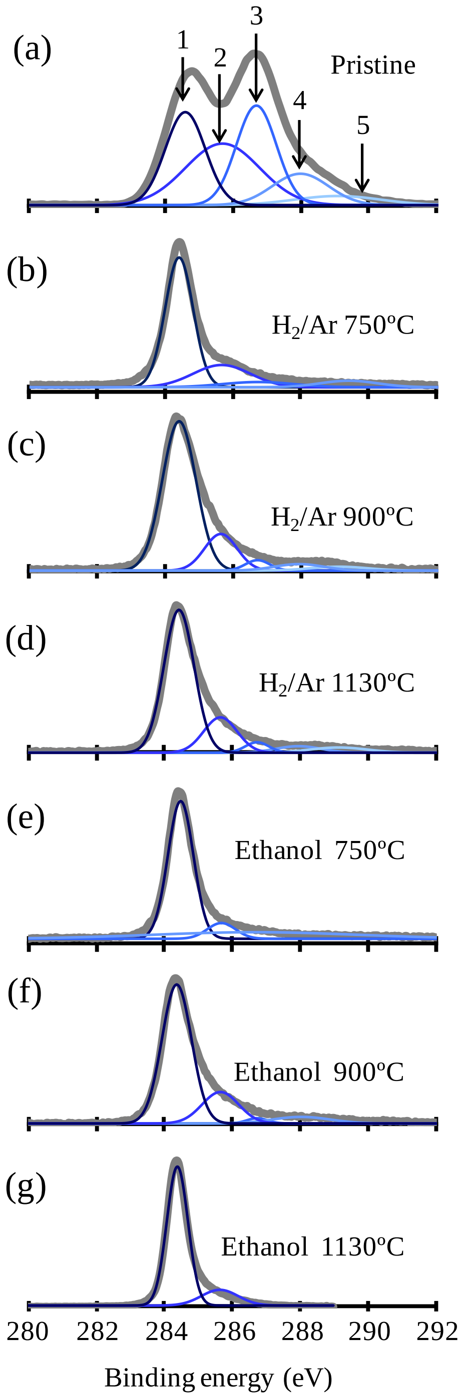

Supplementary Figure S2: ***Chemical bonding states of rGO and pristine GO films using X-ray photoelectron spectroscopy*****.** C 1s spectrum observed from the pristine GO films (Fig. 1(a)) clearly indicates a considerable degree of oxidation with four oxygen-

containing functional groups: deconvolution of C 1s peak of GO displays five peaks at 284.4, 285.7, 286.7, 288.0, and 289.1 eV, which can be assigned to non-oxygenated ring C (1: C-C(H), C=C), 2: C-OH, 3: C-O-C (epoxide), 4: C=O (carbonyl C) and 5: O=C-OH (carboxylate C)[1]. The peak intensities around 286-289 eV in C 1s XPS spectra observed from the rGO films prepared by ethanol and H_2/Ar treatments are much smaller than those in the pristine GO films, indicating efficient removal of the oxygen-containing functional groups, as previously reported[2-4]. Notes that the full width at half maximum (FWHM: 0.7 eV) of the C-C peak observed from rGO films prepared by ethanol treatment at a high temperature of 1130ºC is smaller than that observed from the others (1.1 eV). Since the peak at 284.4 eV is composed of sp^2- and sp^3-hybridized states with slightly different binding energies[5], the small value of FWHM suggests that sp^2-hybridization dominates in the rGO films by a higher process temperature in the ethanol treatment due to efficient structural restoration of the graphitic structure in rGO.

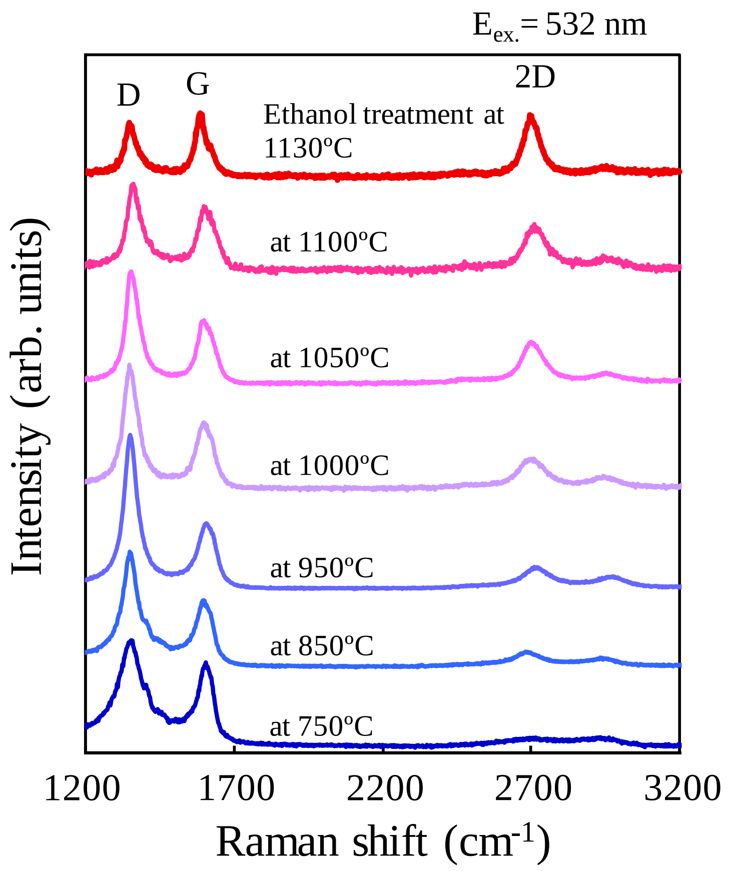

Supplementary Figure S3: ***Structural analysis of rGO films using Raman spectroscopy*****.** Supplemental Fig. S3 shows Raman spectroscopy observed from rGO films prepared by ethanol treatment at several process temperatures. Spectra are normalized by peak intensity of G- band (~1580 cm^{-1}) due to the double degenerate phonon mode at the Brillouin zone center. D-band (~1350 cm^{-1}) mode originates from sp^2-hybridized disordered carbon materials[6]. The 2D-band (~2700 cm^{-1}), which is the overtone of the D-band, is used to determine the number of layers of graphene[7,8]. Thus, the intensity ratio of D- and G-band peaks (I(D/I(G)) is useful to evaluate the structural restoration of rGO[4,9,10].

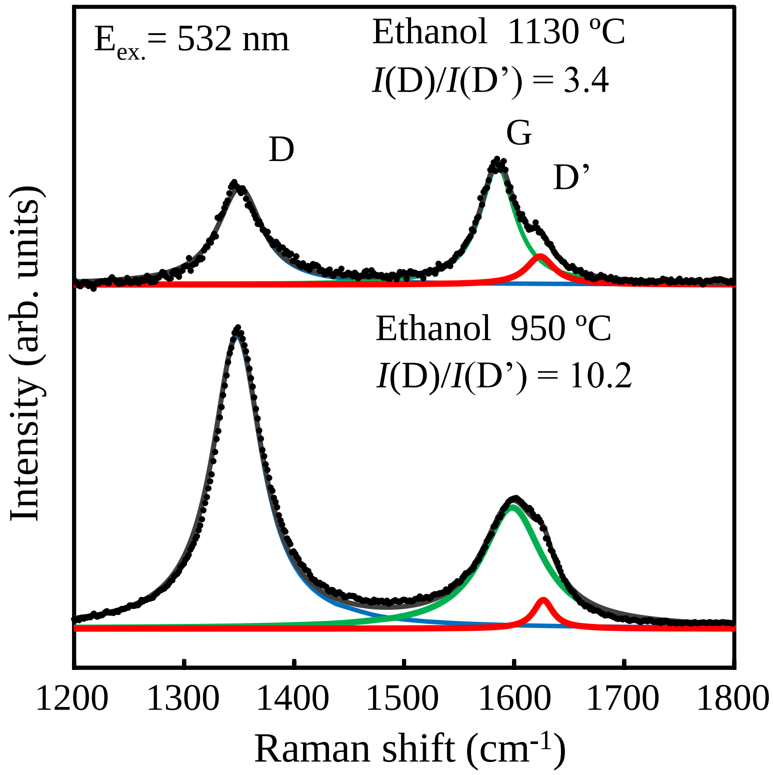

Supplementary Figure S4: ***Raman spectra observed from the rGO films prepared by ethanol treatment at 1130 ºC (upper part) and 950 ºC (lower part), respectively.*** The spectra is fitted using three Lorentzians as indicated by D(blue), G(green) and D’(red). We show that the type of defects which can be defined such as grain boundaries, vacancies and sp^3-defects in the rGO[11,12]. It has been reported that the intensity ratio of the D- and D’-band peaks (I(D)/I(D’))) at ~1620 and ~1350 cm^{-1} originating from the intravalley and intervalley defect-induced resonant scattering processes[13], can be used experimentally to get information on the type of defects in graphene: the I(D)/I(D’) shows a maximum value (~13) for sp^3-defects, it decreases for vacancy-like defects (~7), and it reaches a minimum (~3.5) for boundaries[14,15]. From the analysis of the deconvolution spectra as shown in Fig. S3, we evaluate that the I(D)/I(D’) ~ 3.4 for the rGO films prepared by ethanol treatment at high temperature of 1130 ºC, indicating that the dominant type of defects is not

vacancies and *sp*^{3}-defects, but the boundaries-defects associated with *sp*^{2}-hybridization. On the other hand, the rGO films prepared by ethanol treatment at process temperature below 950 ºC show the *I*(D)/*I*(D') ~ 10.5, suggesting that the dominant types of defects are vacancies and *sp*^{3}-hybridization. This tendency is in good agreement with the result that the FWHM of the peak attributed to C-C in XPS spectra as shown in Fig. S2 decreases with increasing process temperature in ethanol vapor environment.

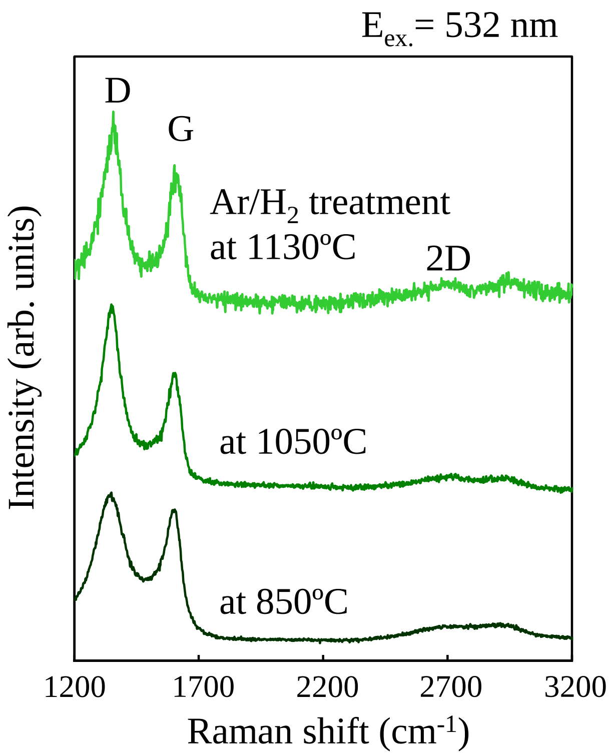

Supplementary Figure S5: ***The process temperature dependence of the crystallinity of the rGO films under the thermal treatment in inert gas.*** Raman spectroscopy observed from the rGO films prepared by Ar/H_2 treatment at various process temperatures.

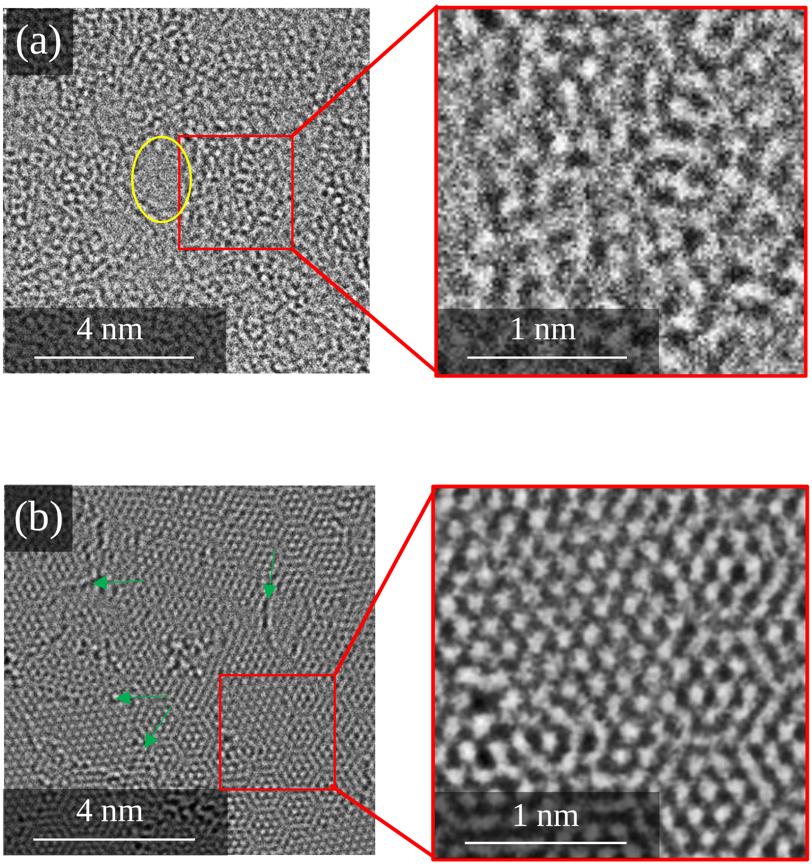

Supplementary Figure S6: ***Transmission electron microscopy (TEM) images obtained from the suspended monolayer rGO sheet prepared by ethanol treatment at (a) 900 ºC and 1100ºC.*** Yellow circle and green arrows indicate the vacancy and domain boundaries. In the TEM image of (a), the many defects such as an amorphous-like π network composed of a mixture of sp^2 and sp^3 structures are observed in the whole region. On the other hand, in the TEM image of (b), the periodic spots are observed indicating improvement of graphene crystallinity. These features are in good agreement with the results of Raman spectra as shown in Fig. 5 and Supplementary Figs. S3, S4 and S5.

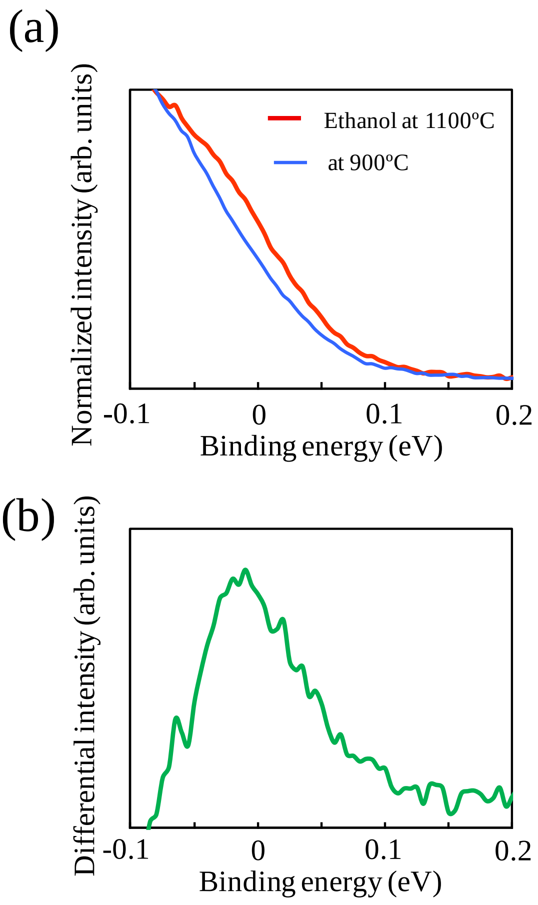

Supplementary Figure S7: ***Process temperature dependence of valence band structures near Fermi level for rGO films.*** (a) Valence band photo-electron spectra observed from rGO prepared by ethanol treatment at 1100°C (red line) and 900°C (blue line), respectively. (b) Their differential spectra (green line) are normalized by integration data of electron dispersion curves from k = -1.42$Å^{-1}$ to 1.42 $Å^{-1}$ at Γ point as a center using photon energy of 95.6 eV, assuming that the number of valence band electrons in the wide range of binding energy from -12 eV to Fermi level is almost the same between rGO films prepared by ethanol treatment at 900 and 1100°C where oxygen-containing functional groups are mostly removed in these rGO films (Supplementary Fig. 1). A study using density functional theory shows that the total DOS in the wide range of binding

energy from -8 to 6 eV is almost the same as that between the pristine graphene and rGO with low oxygen coverage less than 10%[16]. The differential spectrum is obviously seen in positive value near Fermi level, indicating that higher process temperature in ethanol treatment leads to increasing DOS near Fermi level, where they give dominant contribution to the carrier transport mechanism.

Supplementary references

1. Park, S. et al. Aqueous Suspension and Characterization of Chemically Modified Graphene Sheets. *Chemistry of Materials* **20**, 6592-6594 (2008).
2. Akhavan, O. The effect of heat treatment on formation of graphene thin films from graphene oxide nanosheets. *Carbon* **48**, 509-519 (2010).
3. Becerril, H.A. et al. Evaluation of solution-processed reduced graphene oxide films as transparent conductors. *Acs Nano* **2**, 463-470 (2008).
4. Su, C.Y. et al. Highly Efficient Restoration of Graphitic Structure in Graphene Oxide Using Alcohol Vapors. *Acs Nano* **4**, 5285-5292 (2010).
5. Ostrovskaya, L.Y., Dementiev, A.P., Kulakova, I.I. & Ralchenko, V.G. Chemical state and wettability of ion-irradiated diamond surfaces. *Diamond and Related Materials* **14**, 486-490 (2005).
6. Matthews, M.J., Pimenta, M.A., Dresselhaus, G., Dresselhaus, M.S. & Endo, M. Origin of dispersive effects of the Raman D band in carbon materials. *Physical Review B* **59**, R6585-R6588 (1999).
7. Ferrari, A.C. et al. Raman spectrum of graphene and graphene layers. *Physical Review Letters* **97**, 187401-1-4 (2006).
8. Graf, D. et al. Spatially resolved raman spectroscopy of single- and few-layer graphene. *Nano Letters* **7**, 238-242 (2007).
9. Ferreira, E.H.M. et al. Evolution of the Raman spectra from single-, few-, and many-layer graphene with increasing disorder. *Physical Review B* **82**, 125429-1-9 (2010).
10. Krauss, B. et al. Laser-induced disassembly of a graphene single crystal into a nanocrystalline network. *Physical Review B* **79**, 165428-1-9 (2009).
11. Erickson, K. et al. Determination of the Local Chemical Structure of Graphene Oxide and Reduced Graphene Oxide. *Advanced Materials* **22**, 4467-4472 (2010).
12. Gomez-Navarro, C. et al. Atomic Structure of Reduced Graphene Oxide. *Nano Letters* **10**, 1144-1148 (2010).
13. Pimenta, M.A. et al. Studying disorder in graphite-based systems by Raman spectroscopy. *Physical Chemistry Chemical Physics* **9**, 1276-1291 (2007).
14. Eckmann, A., Felten, A., Verzhbitskiy, I., Davey, R. & Casiraghi, C. Raman study on defective graphene: Effect of the excitation energy, type, and amount of defects. *Physical Review B* **88**, 035426-1-11 (2013).
15. Eckmann, A. et al. Probing the Nature of Defects in Graphene by Raman Spectroscopy. *Nano Letters* **12**, 3925-3930 (2012).
16. Huang, H.M., Li, Z.B., She, J.C. & Wang, W.L. Oxygen density dependent band gap of reduced graphene oxide. *Journal of Applied Physics* **111**, 054317-1-4 (2012).
